# Constructing a Teaching Presence Measurement Framework Based on the Community of Inquiry Theory

**DOI:** 10.3389/fpsyg.2021.694386

**Published:** 2021-11-24

**Authors:** Yang Wang, Li Zhao, Shusheng Shen, Wenli Chen

**Affiliations:** ^1^School of Education Science, Nanjing Normal University, Nanjing, China; ^2^National Institute of Education, Nanyang Technological University, Singapore, Singapore

**Keywords:** teaching presence, measurement framework, community of inquiry, reliability and validity, online learning

## Abstract

Given that there is no consensus on a framework for measuring presence in online teaching, this paper focuses on the construction of a reliable measurement framework of teaching presence based on the Community of Inquiry theory. In this study, 408 questionnaires were collected from college students who had online learning experience. Item analysis, exploratory factor analysis, and confirmatory factor analysis were used to analyze the results, which showed that the five-factor framework is in good agreement with the data. The confirmatory factor analysis also demonstrated a good model fit of the correlated five-factor teaching presence framework. Therefore, the teaching presence measurement framework consisting of design and organization, discourse facilitation, direct instruction, assessment, and technological support, can serve as an effective tool to support teaching presence measurement and to provide guidance for instructors’ online teaching.

## Introduction

Online learning has dramatically increased in recent years. As such, online education has been applied in all education stages including formal and informal education (Martine et al., 2020). Online learning has brought convenience to teaching and learning without the restraint of time and space. However, the quality of online learning needs to be improved (Chen et al., 2021; Wang et al., 2021). Lee and Recker (2021) proposed that online learning quality depended not only on online learning resources but also on instructors’ teaching presence. Teaching presence determines students’ learning efficiency (Caskurlu et al., 2020). It is a link between curriculum content and learners. Due to physical separation, face-to-face communication and instant feedback are reduced in online learning. It seems that the demand for teaching presence has weakened. However, in the online learning environment, the requirements for teaching presence are higher (Wang and Liu, 2020).

Teaching presence can be interpreted as the visibility of the instructor, which influences students’ participation and engagement (Caskurlu et al., 2020). It gives instructors guidance on course design and organization to facilitate students’ learning. Teaching presence is taken as a useful tactic in the process of online learning (Akyol et al., 2009). Specifically, the establishment and maintenance of a community of inquiry require a comprehensive teaching presence (Shea et al., 2006; Akyol et al., 2009). It is teaching presence that enhances students’ cognitive and social presence (Zhang et al., 2016; Law et al., 2019). However, in the online environment, it is not necessary that the instructor should respond to every student’s post, but the instructor acting as a mediator and guide is helpful for students’ discussion. That is, instructors are expected to design effective online activities to support students’ high-level cognitive interactions. For example, Wang and Liu (2020) compared three courses and found that the design and facilitation improved students’ interaction and knowledge construction. Caskurlu et al. (2020) tested the relationship between learning outcomes and teaching presence and found that there was a strong correlation between teaching presence and students’ perceived learning as well as their satisfaction. Preisman (2014) also supported that the instructor plays an essential role in facilitating students’ online learning. Designing a well-structured online course is of great significance for the instructor. Similarly, Szeto (2015) found that the expected learning outcomes are less dependent on the social and cognitive presences than on the teaching presence. That is, studies support that teaching presence is essential to an online community of inquiry. The components of teaching presence have therefore become a focus of online teaching research.

Currently, four methods can be adopted to measure teaching presence. Anderson et al. (2001) hold that teaching presence is mainly composed of two elements: instructional design and organization, and facilitating instruction. Akyol et al. (2009) support that teaching presence consists of three elements: design and organization, discourse facilitation, and direct instruction. This interpretation is also supported by Caskurlu (2018). Shea and Bidjerano (2010) took a step further and added “assessment” as an important element, while also redefining the confusing elements of “discourse facilitation” and “direct instruction”. Shea et al. (2010) supported that teaching presence in online learning environments consists of design and organization, discourse facilitation, direct instruction, and assessment. There are also many researchers who support that teaching presence is a general concept (Coppola et al., 2002; LaPointe and Gunawardena, 2004; Arbaugh and Hwang, 2006). Despite there are many studies on the teaching presence, its measurement framework is still to be explored. Herein, the purpose of this study is to explore the framework of teaching presence.

## Literature Review

### Community of Inquiry

The Community of Inquiry (CoI) theory was proposed by Garrison et al. (2001) to illuminate the multifaceted components of teaching and learning (Garrison et al., 2000). The CoI theory supports that learners’ social, cognitive, and teaching presence are three basic factors associated with their perceived learning. Social presence is the level of learners’ recognition of the learning environment and the learning group. Cognitive presence is the degree of learners’ meaning construction through continuous reflection and discourse (Shea et al., 2014). Teaching presence can be defined as a means of designing, facilitating, and directing cognitive and social processes to achieve personal and educational value (Anderson et al., 2001). Teaching presence, as one of the key element in CoI, is highly related to social and cognitive presence (Garrison et al., 2010). It is aimed at designing, facilitating, and directing social and cognitive presence to achieve expected learning outcomes (Anderson et al., 2001). While learning online, teaching presence determines students’ learning satisfaction (Khalid and Quick, 2016; Kyei-Blankson et al., 2019), performance (Arbaugh, 2008), and engagement behaviors (Zhang et al., 2016). Thus, teaching presence is an important factor determining online learning efficiency (Gurley, 2018).

### Teaching Presence

There have been many studies on online teaching presence, most of which have focused on the relationship between online teaching presence and learning engagement (Zhang et al., 2016), students’ interactions and collaborative knowledge construction (Wang and Liu, 2020), and students’ learning satisfaction (Caskurlu et al., 2020). For example, teaching presence is not the same as traditional teaching presence in a face-to-face classroom (Gurley, 2018). Instructors must communicate effectively with students despite being separated from them by time and place. It is supported that students and teachers play essential roles in teaching presence, with teachers playing the main role in constructing teaching presence (Wang and Liu, 2020). Hence the teacher’s teaching presence in online environments was explored further in this study to give instructors practical suggestions to improve their online teaching.

Although teaching presence is important, there is not a consensus on its measurement. Garrison et al. (2000) proposed the Community of Inquiry theory and scaled teaching presence with three dimensions: teaching management, constructing understanding, and direct instruction, based on existing studies conducted in western countries. Shea et al. (2005) explored the structure of teaching presence through factor analysis in the United States and found that two factors (i.e., design and organization, and directed facilitation) fit the data well. They proposed that direct instruction is a factor of facilitation and may not be an indicator of teaching presence. To further explore the structure of teaching presence, Shea et al. (2006) examined the two-factor model consisting of discourse facilitation and direct instruction through a Principal Component Analysis (PCA) in the United States. Arbaugh and Hwang (2006) investigated Master of Business Administration (MBA) students in a Mid-Western United States university and found that the three-factor model (i.e., instructional design and organization, facilitation, and direct instruction) fit the data well through confirmatory factor analysis. Caskurlu (2018) performed a confirmatory factor analysis at a university in the United States and also supported that teaching presence can be scaled with three dimensions: design and organization, facilitation, and direct instruction. However, they found that there may be some overlaps between direct instruction and facilitation. Given there is no consensus on teaching presence measurement, this study explored a measurement framework to improve online teaching.

### Research Purpose

In the context of online learning, the online teaching platform is the foundation. Technological support is of great significance to the development and success of online teaching. Therefore, it is believed that technological support is also an important role of instructors in the online teaching presence. It is supported that three aspects can be improved in the online teaching presence measurement research.

First, the attention to the technological support is insufficient. The instructor not only acts as the designer, facilitator, instructor, and evaluator, but also as the technological supporter in the online community of inquiry. The widely accepted teaching models, Pedagogy-Society-Technology (PST) and the Technological Pedagogical and Content Knowledge (TPACK), both emphasize the important role of technology in teaching. PST supports that education is always a unique combination of technological, social, and educational contexts and affordances (Kirschner et al., 2004). Among them, pedagogy is the teaching practice to achieve specific teaching objectives, and mainly includes teaching content, activities, and assessment. Social interaction refers to activities that promote learners’ interaction, including the interactive environment, tools, and interactive rules. Technological support represents the extent to which technology supports learning, including the usefulness, usability, and ease of use of technology. The model proposes that online teaching will become a castle in the air without technological support. The CoI and PST both emphasize that technological support is necessary for a meaningful online learning experience. TPACK was built based on Shulman (1986) model to describe how teachers perceive educational technologies. With the support of technology, the elements of Pedagogical Content Knowledge (PCK) interact with each other to produce effective teaching (Koehler and Mishra, 2009). The TPACK model defines teaching competencies from three aspects: technological, pedagogical, and content knowledge. Technological knowledge refers to the knowledge that enables a person to accomplish occupational tasks using information technology. TPACK emphasizes the role of technology in teaching, and holds that technology and teaching are mutually integrated. However, technological support is not sufficiently considered in the Community of Inquiry model.

Second, the definition of “design and organization” needs to be expanded. Design and organization were initially described as pre-class activities including curriculum structure, collaborative and individual activities, timetables, and expectations (Anderson et al., 2001). Although most design takes place before classes, the second component, “organization,” represents the arrangement of scattered people or things in a systematic way to achieve the same teaching objective. It consists of the rules and procedures of inquiry activities in online communities, including not only the design and organization before discussion activities, but also the design, organization, and management during and after activities.

Third, the scope of research needs to be expanded (Caskurlu, 2018). The application environment of teaching presence was online discussion when it was first proposed. However, a great deal of teaching support is also necessary and observed in areas besides online discussions. To understand the role of teaching presence, all observable teaching support should be analyzed. The support mainly includes participating in discussions, answering students’ questions, providing related materials, arranging activities, and other teaching practices related to the course.

Hence, this study constructed a model to explain the online teaching presence measurement framework based on the CoI theory, which consists of five factors: design and organization, discourse facilitation, direct instruction, assessment, and technological support. The purpose of the study was to (1) test whether the five-dimensional model is a reliable tool for the measurement of teaching presence; and (2) explore the internal relationships among the five factors.

## Methods

### Research Design

#### Preliminary Development of the Teaching Presence Measurement Framework

Given the three problems of existing teaching presence measurement frameworks mentioned above, a teaching presence measurement framework including the following five dimensions: design and organization, discourse facilitation, direct instruction, assessment, and technological support, was developed. The design and organization, and discourse facilitation are measured with five and eight indicators (Anderson et al., 2001; Akyol et al., 2009). The direct instruction and assessment are scaled with five and six indicators (Shea et al., 2010). The technological support is scaled with six indicators (Shea et al., 2010; Wang and Liu, 2020). Finally, there are 30 items in the questionnaire to measure instructors’ teaching presence. Each item was measured using a 5-point Likert scale (ranging from *strongly agree to strongly disagree*, as shown in Table 1). To further explore the importance ranking of the five dimensions of teaching presence, a question on the perceived importance of the five dimensions was added at the end of the questionnaire.

**TABLE 1:** Items of the five-dimensional teaching presence scale.

**Dimension**	**No.**	**Indicator**	**Code**	**Source**
Design and organization (DO)	1	The teacher communicated essential course outcomes, e.g., goals, strategies, schedule, expectations, and rubrics	DO1	Akyol et al. (2009)
	2	The teacher provided instructions on participating in course activities, e.g., illuminating strategies to fulfill assignments successfully	DO2	Anderson et al. (2001)
	3	The teacher communicated accurate schedule of learning activities to guide students keep pace with each other	DO3	
	4	The teacher helped students understand the rules of online learning behaviors	DO5	
	5	The teacher provided explanation for the significance of assignment	DO6	
Discourse facilitation (DF)	6	The teacher helped to examine areas of agreement and disagreement to facilitate students’ learning	FD1	Akyol et al. (2009)
	7	The teacher helped to reach agreement	FD2	Anderson et al. (2001)
	8	The teacher encouraged and enhanced contributions	FD3	
	9	The teacher set an inquiry environment	FD4	
	10	The teacher facilitated students’ discussion	FD5	
	11	The teacher evaluated the effectiveness of the learning process	FD6	
	12	The teacher refocused on specific topics to be discussed	FD7	Shea et al. (2010)
	13	The teacher summarized discussions	FD8	
Direct instruction (DI)	14	The teacher offered useful examples of analogies	DI1	Shea et al. (2010)
	15	The teacher provided helpful explanations	DI2	
	16	The teacher delivered informative presentations	DI3	
	17	The teacher clarified information provided	DI4	
	18	The teacher mentioned external materials explicitly	DI5	
Assessment (AS)	19	The teacher provided formative feedback for discussion	AS1	Shea et al. (2010)
	20	The teacher offered formative feedback for coursework	AS2	
	21	The teacher provided summary feedback for discussion	AS3	
	22	The teacher offered summary feedback for assignments	AS4	
	23	The teacher asked students for formative feedback of curriculum design and activities	AS5	
	24	The teacher asked students for a summary feedback of curriculum design and activities	AS6	
Technological support (TS)	25	The teacher made full use of technology in teaching	TS1	Shea et al. (2010)
	26	The teacher diagnosed technical problems that students may face before class	TS2	Stein and Wanstreet (2017)
	27	The teacher chose the appropriate media according to the expected learning results	TS3	
	28	The teacher used different medias to promote different learning styles	TS4	
	29	The teacher edited and updated distributed learning resources	TS5	
	30	The teacher respected for intellectual property rights	TS6	

#### Pretest and Formal Test

To ensure the validity of the measurement framework, five educational technology experts examined the items before the questionnaire was further tested. To ensure the popularity, accuracy, and objectivity of the items, the questionnaire was pretested by 24 online learners, and the presentation of the items was improved according to their feedback. Finally, a questionnaire consisting of 35 items was constructed, including four items on personal background information, five on design and organization, eight on discourse facilitation, five on direct instruction, six on assessment, six on technological support, and one on the perceived importance of the five dimensions of teaching presence.

### Data Collection

In November 2018, the questionnaire was distributed to learners majoring in educational technology from four universities in central, western, and eastern regions of China through an online social communication platform. They enrolled in the same online training consisting of several courses at one platform conducted by one instructor from a university in central China. At the end of the training, students were administered a questionnaire on their perceived teaching presence in the same training courses. Participants were told to answer questions according to their online learning experience. After completing the questionnaire, participants were entered in a lottery to win a random amount of money ranging from 10 to 50 RMB as an incentive. Participants should answer all 35 questions before submitting the questionnaire. In the introduction of the questionnaire, the purpose, duration, and anonymity of the survey were explained. A total of 416 questionnaires were collected. Three experimenters who were familiar with the items answered the questionnaire. They felt that it should take at least 30 s to complete. The average answer time of the collected samples was 219.88 s. Thus, eight questionnaires submitted in less than 30 s were deleted, leaving 408 valid questionnaires.

### Measurements

To construct a teaching presence measurement framework and to verify its effectiveness, the following measurements were conducted. The samples were randomly divided into two groups, with 204 in each (Yurdakul et al., 2012). The first sample was subjected to exploratory factor analysis (Vogel et al., 2009). Confirmatory factor analysis was applied to the second sample. First, exploratory factor analysis (EFA) was conducted on the data of 204 questionnaires, and the results of principal component analysis were used to further improve the questionnaire. Second, confirmatory factor analysis (CFA) was conducted on the remaining 204 questionnaires to verify the results. Finally, item analysis was performed on all samples to test the suitability and differentiation of the questions. Data were analyzed using SPSS 25.0 and AMOS 24.0.

## Results

### Exploratory Factor Analysis

The EFA was conducted using SPSS 24.0, and factors were rotated with the maximum variance method. The KMO value was 0.950 (higher than 0.9), and the Bartlett sphericity test showed that there was a correlation between variables (x2 = 3873.077; *p* = 0.000 < 0.001), demonstrating that these data were applicable for exploratory factor analysis.

To test the validity of the measurement dimensions, the principal component extraction (PCA) method was used to extract factors, and five factors were finally obtained. Although the factors were found to be correlated after the preliminary analysis, the oblique rotation method was better. However, since the purpose of this study was to replicate the analysis, Kieffer (1998) suggests that researchers should use two strategies for exploratory factor analysis. When there is no difference between the results of the orthogonal and oblique rotations, the analysis results of the orthogonal rotations can be used. Therefore, the maximum variance orthogonal rotation method and the optimal skew are used for exploratory factor analysis. The results of the two analyses were similar. Therefore, this paper presents the results of the maximum variance orthogonal rotation method. FD3, DI1, TS1, and TS2 were removed as their maximum factor loadings were not in their measurement dimension (Conway and Huffcutt, 2016). The maximum variance rotation method was used to determine the factors’ interpretability. The result is shown in the component transformation matrix (see Table 2). The standardized factor loading of each factor was greater than 0.5, indicating that the factors demonstrated good interpretability (Fabrigar et al., 1999).

**TABLE 2:** Teaching presence measurement factor analysis (N Sample 1 = 204).

**Items**	**1**	**2**	**3**	**4**	**5**
DO1				0.726	
DO2				0.754	
DO3				0.597	
DO4				0.601	
DO5				0.580	
FD1	0.616				
FD2	0.621				
FD4	0.647				
FD5	0.563				
FD6	0.659				
FD7	0.610				
FD8	0.616				
DI2					0.766
DI3					0.768
DI4					0.683
DI5					0.504
AS1		0.541			
AS2		0.662			
AS3		0.685			
AS4		0.747			
AS5		0.609			
AS6		0.581			
TS3			0.678		
TS4			0.691		
TS5			0.653		
TS6			0.659		

The principal component analysis was applied to extract factors, and maximum variance rotation was used for the EFA. The factors with an eigenvalue higher than 1 were picked. Items with less than 0.4 on factor loading and inconsistent content were removed through the multiple orthogonal rotations (Zhao et al., 2021a). There were 26 items with eigenvalues greater than 1 and independent factor loadings greater than 0.5 which were retained (Fabrigar et al., 1999). Finally, five factors were selected, with a cumulative variance contribution of 65.744% (Conway and Huffcutt, 2016). The eigenvalues and cumulative variance contributions of the five factors are shown in Table 3.

**TABLE 3:** The eigenvalues and contribution rates of the five factors in the model.

**Component**	**Eigenvalue**	**Percentage of variance**	**Cumulative variance contribution rate**
1	12.293	47.281%	47.281%
2	1.535	5.904%	53.185%
3	1.172	4.508%	57.693%
4	1.061	4.080%	61.773%
5	1.032	3.971%	65.744%

### Confirmative Factor Analysis

The first-order CFA is applied to determine the reliability, convergence, and identifiability of the framework in this study. The CFA is used to explore the relationships among factors, and then to build the online teaching presence measurement framework.

#### Fitting Validity Analysis for Framework

**FIGURE 1:**
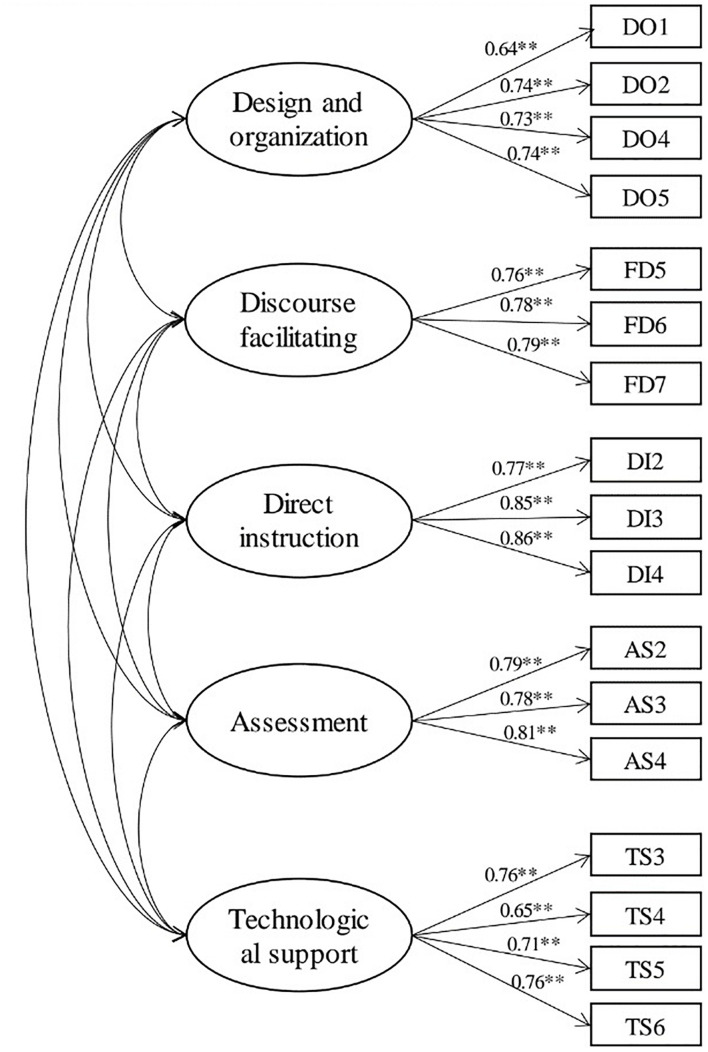
First order confirmatory factor analysis model. **p < 0.01.

In the first-order confirmatory factor analysis (see Figure 1), the item with a standardized loading less than 0.5 has to be removed (Hair et al., 2014). To examine the model fit, the absolute and relative fitting indexes were calculated. In this study, the chi-square/df was 1.183. The RMSEA was 0.030 (<0.08) (Liu et al., 2021). The values of GFI and AGFI were 0.933 and 0.906 (>0.9) (Foster et al., 1993). The values of NFI, CFI, and RFI were 0.932, 0.989, and 0.915 (>0.9) (Hair et al., 2014).

Given the model indexes in Table 4, such as the chi-square/df, RMSEA, GFI, AGFI, NFI, CFI, and IFI, all were acceptable. DO3, DF1, DF2, DF4, DF8, DI1, DI5, AS1, AS5, and AS6 were deleted. The 17 remaining items were used for further analysis, including design and organization (4 items), discourse facilitation (3 items), direct instruction (3 items), assessment (3 items), and technological support (4 items).

**TABLE 4:** Framework fitting index.

**Type**	**Fitting index**	**Threshold**	**Values**	**Results**
Absolute fit index	Chi-square/df	<3	1.183	Supported
	RMSEA	<0.08	0.030	Supported
	Goodness-of-fit index (GFI)	>0.8	0.933	Supported
	Adjust fitness index (AGFI)	>0.8	0.906	Supported
Relative fit index Incremental fit index	Normed fitness index (NFI)	>0.9	0.932	Supported
	Non-normalized fitness index (NNTI/TFI)	>0.9	0.986	Supported
	Comparative fitness index (CFI)	>0.9	0.989	Supported
	Incremental fitness index (IFI)	>0.9	0.989	Supported
	Relative fitness index (RFI)	>0.9	0.915	Supported
Streamlining fit index Parsimonious fit index	Simplify the specification fitness index (PNFI)	>0.5	0.747	Supported
	Streamlining fitness indicators (PGFI)	>0.5	0.665	Supported

#### Convergence Validity Analysis for Framework

Specifically, the composite reliabilities (CR) of all items were higher than 0.80 (>0.7) which is considered to be good (Hair et al., 2014). It shows that the dimension has a convergence effect if the Average Variance Extracted (AVE) exceeds 0.5 (Fornell and Larcker, 1981; see Table 5). That is, the framework in this study is reasonable and the questionnaire has high validity.

**TABLE 5:** Results of confirmatory factor analysis.

**Latent variable**	**Measure item**	**Standardized factor loading**	**CR**	**AVE**
Design and organization (DO)	DO1	0.639	0.8063	0.5109
	DO2	0.742		
	DO4	0.734		
	DO5	0.739		
Discourse facilitation (DF)	DF5	0.757	0.8167	0.5977
	DF6	0.776		
	DF7	0.786		
Direct instruction (DI)	DI2	0.773	0.8668	0.6848
	DI3	0.848		
	DI4	0.859		
Assessment (AS)	AS2	0.792	0.8347	0.6274
	AS3	0.777		
	AS4	0.807		
Technological support (TS)	TS3	0.759	0.8135	0.5226
	TS4	0.764		
	TS5	0.651		
	TS6	0.712		

*CR represents Composite reliability; AVE represents Average variance extracted.*

#### Reliability Analysis of the Scale

The reliability of the questionnaire was scaled with the Cronbach’s alpha and composite reliabilities. After exploratory factor analysis and confirmatory factor analysis, DO1, DO2, DO4, DO5, FD5, FD6, FD7, DI2, DI3, DI4, AS2, AS3, AS4, TS4, TS5, and TS6 were retained. The Cronbach’s alpha of adjusted scale was 0.930 and the Cronbach’s alpha of DO, DF, DI, AS, and TS were 0.804, 0.817, 0.866, 0.834, and 0.812, respectively. The composite reliabilities of DO, DF, DI, AS, and TS were 0.8063, 0.8167, 0.8668, 0.8347, and 0.8135, respectively, which were considered to be high by Bagozzi and Yi (1988).

#### Discriminant Validity Analysis for Framework

The structural discriminant validity analysis of the tool is shown in Table 6. In general, the square root of AVE for each dimension should be higher than the absolute value of the Pearson correlation coefficient between the two dimensions, which can be identified as discriminant validity. This result demonstrated that the framework had good discriminant validity (Schumacker and Lomax, 2016).

**TABLE 6:** Correlation coefficient matrix and square roots of AVE.

**Construct**	**DO**	**DF**	**DI**	**AS**	**TS**
DO	0.715				
FD	0.607**	0.773			
DI	0.547**	0.620**	0.828		
AS	0.563**	0.636**	0.625**	0.792	
TS	0.521**	0.655**	0.593**	0.630**	0.723

*The data at the diagonal is the square root of AVE, and the rest of the data is Pearson correlation coefficient.*

**p < 0.01.

The five-factor framework has good convergence validity and discriminatory validity through the first-order confirmatory factor analysis. That is, the model can be used to interpret the data.

### Item Analysis

The purpose of item analysis is to test the appropriateness and discrimination of questions. Item analysis examines two main aspects: the first aspect is the decisive value, and the second aspect is the correlation coefficient between question items and the total score of the dimensions. That is, an independent samples *t*-test was conducted for question items in the high group versus the low group. The top 27% and bottom 27% in the sample of 408 participants in the item analysis were defined as the high and low groups, referring to Aridag and Yüksel (2010). Items that did not reach a significant difference between the two groups were deleted.

Specifically, questions with dimensional Pearson correlation coefficients less than 0.4 and questions with standardized factor loadings less than 0.45 needed to be deleted (Kim, 2014). Based on these criteria, after item analysis of the questionnaire, the decisive values of the remaining items were all greater than 0.3, and the total correlation coefficient between items and questions was greater than 0.4. Therefore, through the item analysis, the remaining 17 questions met the criteria.

### The Relationship of the Five Factors in the Framework

Based on the findings and the Community of Inquiry framework proposed by Garrison et al. (2001), this study constructed a teaching presence measurement framework. The results show that instructors’ teaching presence can be measured according to five aspects: design and organization, discourse facilitation, direct instruction, assessment, and technological support. There are correlations among these five factors. Perceived importance is scored from 1 to 5, with 1 being the most important and 5 being the least important. The results of the perceived importance question were processed in reverse. Therefore, the higher the score, the more important the factor. The result is shown in Table 7. The learners’ perceived importance of the five dimensions of teaching presence is: design and organization > discourse facilitation > direct instruction > assessment > technological support.

**TABLE 7:** The perceived importance of the five dimensions of teaching presence.

**Dimensions**	**Average**	**Rank**
Design and organization (DO)	3.65	1
Discourse facilitation (DF)	3.15	2
Direct instruction (DI)	2.69	3
Assessment (AS)	2.02	4
Technological support (TS)	1.81	5

**FIGURE 2:**
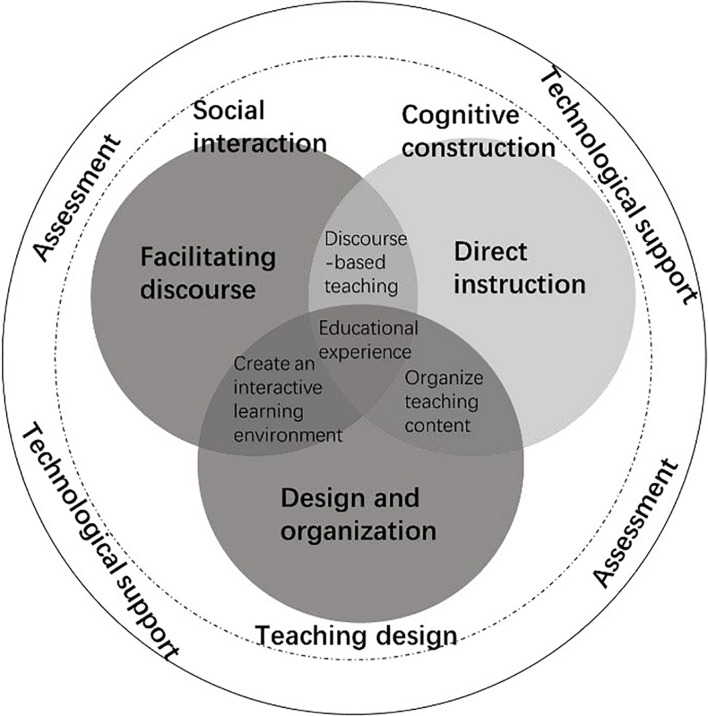
Teaching presence measurement model.

Therefore, it can be concluded that the design and organization, discourse facilitation, and direct instruction are three key elements of the framework (see Figure 2). The external teaching environment is created by instructors’ technological support and assessment. The perceived importance of technological support is higher than that of the assessment which indicated that the technological support in online teaching was essential. Discourse facilitation is aimed at promoting learners’ social interaction. Direct instruction is aimed at promoting learners’ cognitive construction. Design and organization are adopted to design teaching activities. Discourse facilitation and instruction are used to construct discourse-based teaching. Design and organization, and discourse facilitation are to create an interactive learning environment. Design and organization, as well as direct instruction are applied to organize teaching content. Students’ interaction and collaborative knowledge construction can be facilitated with assessment and technological support in the whole learning process. That is, the teaching presence measurement framework can provide a reference for instructors’ online teaching.

## Discussion

Online teaching presence is a comprehensive reflection of instructors’ online teaching competencies. Thus, the construction of the teaching presence measurement framework in the online community of inquiry can not only provide a reference for online teaching assessment but can also promote teaching by assessment. It provides instructors with practical suggestions from the perspectives of design and organization, discourse facilitation, direct instruction, technological support, and assessment. Furthermore, learners’ perceived importance of the five dimensions of teaching presence also indicates that instructors should pay attention to online learning activities design, discourse facilitation in online discussions, direct instruction, technological support, as well as learning assessment.

The measurement framework constructed in this study differs from that of Shea et al. (2010) who proposed that teaching presence in online learning environments consisted of design and organization, discourse facilitation, direct instruction, and assessment. Two possible reasons could be used to explain this discrepancy. One may be the different research backgrounds. This study was conducted in China, whereas that of Anderson et al. (2001) was conducted in the United States Chinese and western students’ expectations of teaching and learning may differ. Chinese students prefer learning independently and tend to be modest and emphasize the importance of order and respect for authority (Sit, 2013), whereas Western students tend to communicate with others and stress egalitarianism, individual development, and cooperation (Elbers, 2010). That is, more importance is attached to discourse facilitation in western online education, while more importance is attached to direct instruction and technological support in Chinese online education. Another reason may be the different online learning environments in Chinese and western countries. For example, platform construction and video lecture design are emphasized in Chinese online education (Zhao et al., 2021b), whereas reading, discussion, collaboration, and reflection are dominant online learning activities in western countries (Misko et al., 2004). For this reason, the perceived technological support was relatively higher in China and hence the technological support becomes a significant dimension of the teaching presence measurement framework. It does not mean, however, that teachers should invest more in direct instruction and technological support. Since the perceived importance of the five dimensions of teaching presence supports that design and organization and discourse facilitation are key to the community of inquiry, teachers could improve their teaching practice based on the online teaching presence framework.

## Conclusion

Since there is no consensus on the online teaching presence measurement, it is valuable to explore the latent factors of teaching presence to examine whether they provide a reliable solution for the measurement of online teaching presence. In this study, the item analysis, EFA, and CFA were applied to construct a five-factor teaching presence framework. This framework consists of design and organization, discourse facilitation, direct instruction, assessment, and technological support. It can serve as an effective tool to support teaching presence measurement and to provide guidance for instructors’ online teaching.

### Implications

There are two contributions made by this study. On one hand, this study carried out research on the teaching presence measurement method. It has been reported that there are some differences in online teaching in China and western countries (Liu and Meng, 2009). That is, studies in western countries may not satisfy the needs of Chinese online learners. As such, it is valuable to further explore the teaching presence measurement framework in China. It can also be a support tool for other Asian countries like China. On the other hand, the results of the item analysis, EFA, and CFA support the reliability and validity of the five-factor framework which indicates that the online teaching presence measurement framework consists of design and organization, discourse facilitation, direct instruction, assessment, and technological support.

### Limitations and Future Study

The present study contributes to the field. However, there are still limitations to this study that should be noted. For example, the sample in this study was from several provinces of China selected by random sampling, which cannot cover all the universities in the whole country. More and larger representative samples will be needed in the future to assess the extent to which the findings are applicable to other population groups and other countries to confirm the conclusion of the study. Additionally, all the courses in this study were instructed by the same teacher which limits the application of more robust analytic methods. Hence, in the future study, it would be valuable to further explore the teaching presence measurement framework based on data collected from multiple teachers, which allows us to adopt the more appropriate multilevel confirmatory factor analysis method given that the items, despite being rated by students, measure the traits of the teachers (Stapleton et al., 2016a,b).

## Ethics Statement

Ethical review and approval was not required for the study on human participants in accordance with the local legislation and institutional requirements. The experimental data they provided was anonymous and would not be of any commercial use or influence their final course scores. All the students agreed to participate in the study. Written informed consent from the college students/participants was not required to participate in this study in accordance with the national legislation and the institutional requirements.

## Author Contributions

All authors contributed equally to the conception of the idea, implementing and analyzing the experimental results, and writing the manuscript and read and approved the final manuscript.

## Conflict of Interest

The authors declare that the research was conducted in the absence of any commercial or financial relationships that could be construed as a potential conflict of interest.

## Publisher’s Note

All claims expressed in this article are solely those of the authors and do not necessarily represent those of their affiliated organizations, or those of the publisher, the editors and the reviewers. Any product that may be evaluated in this article, or claim that may be made by its manufacturer, is not guaranteed or endorsed by the publisher.
